# Mediterranean diet adherence and risk of kidney stones: Insights from a population-based study

**DOI:** 10.1097/MD.0000000000044653

**Published:** 2025-09-19

**Authors:** Si-yu Chen, Li Wang, Jian-wei Yang, Kang-yu Wang, Xiao-ran Li, Li Yang

**Affiliations:** a Department of Urology, Gansu Province Clinical Research Center for Urinary System Disease, The Second Hospital & Clinical Medical School, Lanzhou University, Lanzhou, China; b Institute of Urology, Gansu Province Clinical Research Center for Urinary System Disease, The Second Hospital & Clinical Medical School, Lanzhou University, Lanzhou, China.

**Keywords:** alternate Mediterranean diet score, dietary, kidney stone, NHANSE

## Abstract

This study explored the relationship between the alternative Mediterranean Diet (aMED) score and the prevalence of kidney stones (KSD) among adults in US. This cross-sectional analysis utilized data from the National Health and Nutrition Examination Survey spanning 2007 to 2018. The study population comprised adults aged 20 years and older who provided comprehensive dietary recall information and detailed histories of KSD. Adherence to the Mediterranean diet was quantified using the aMED score, which was divided into 4 quartiles for comparison. To evaluate the association between aMED scores and KSD prevalence, we employed weighted multivariable logistic regression, conducted restricted cubic spline analyses, and performed subgroup investigations. Of the 28,059 participants, 10.08% reported a history of KSD. The weighted mean age (95% CI) was 47.97 years (47.49, 48.45). Males comprised 52.49% (50.08%, 54.90%) of the sample, while females made up 47.51% (45.32%, 49.70%). The restricted cubic spline analysis revealed a negative linear association between the aMED score and the likelihood of KSD development. In a fully adjusted model, individuals in the highest quartile of aMED scores (Q4) were found to have a significantly lower risk of KSD compared to those in the lowest quartile (Q1), with an odds ratio of 0.771 (95% CI: 0.616–0.966, *P* = .024). An increased aMED score was associated with a lower prevalence of KSD in U.S. adults. This cross-sectional study provides observational evidence suggesting a potential link between dietary patterns and KSD prevalence, which may inform future dietary intervention research.

## 1. Introduction

Globally, kidney stone disease (KSD) affects an estimated 10% to 12% of individuals, with a significant increase in cases observed in recent years.^[1]^ In the U.S., prevalence has surged from 3.8% in the 1970s to around 10% today, disproportionately affecting males and the elderly.^[2]^ Dietary habits, obesity, and metabolic conditions like diabetes and hypertension are contributing factors. Nearly 50% of individuals who form stones experience recurrence within 5 to 10 years, emphasizing the necessity for effective preventive strategies.^[3]^

With the rising incidence of KSD and their significant impact on public health, there is a need to find modifiable risk factors to modulate the incidence of the disease. Dietary habits have emerged as key determinants in KSD formation. An inflammatory diet has been associated with an increased incidence of KSD,^[4]^ whereas a vegetarian diet has been linked to a decreased incidence.^[5]^ The Mediterranean diet (MED) has been shown to lower the risk of cardiovascular diseases, type 2 diabetes, and various cancers.^[6]^ Despite extensive research on the health benefits of the MED, its role in KSD prevention remains relatively underexplored.

The Adherence to the MED (aMED) score is a widely used tool for quantifying adherence to this dietary pattern. Higher aMED scores reflect better adherence and are generally associated with favorable health outcomes.^[7]^ However, the potential protective effect of a higher aMED score on KSD formation requires further investigation, particularly in populations like U.S. adults, whose dietary habits differ significantly from those of the Mediterranean region. Understanding this relationship could provide valuable insights for dietary interventions aimed at KSD prevention.

## 2. Methods

National Health and Nutrition Examination Survey assesses the nutritional and health status of U.S. children and adults using stratified, multistage sampling^[8]^(www.cdc.gov/nchs/nhanes/). No additional ethical application is required for the study.

### 2.1. Study population

**Figure 1.**
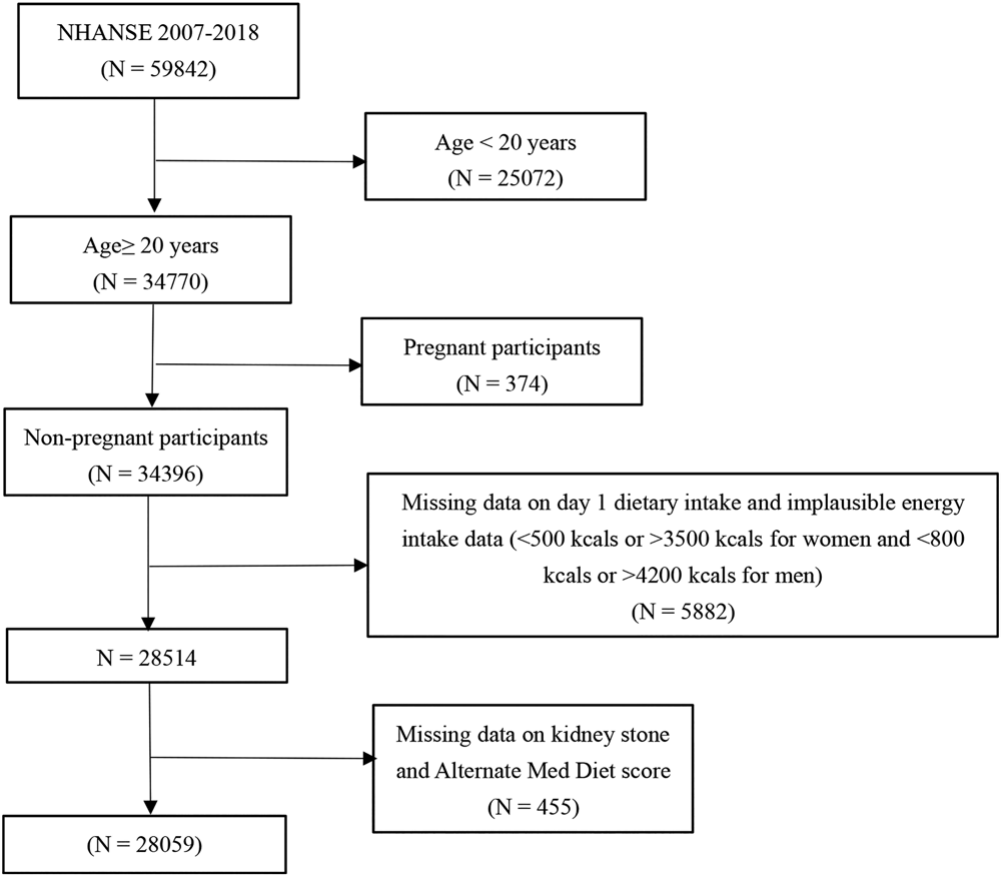
The flowchart of the study.

Data from the NHANES from 2007 to 2018 were initially analyzed, including a total of 58,942 participants. The following groups were excluded from the study: participants age ≥ 20 (n = 25,072), pregnant individuals (n = 374), participants with missing or implausible dietary intake data on Day 1 (n = 5882) (defined as < 500 kcal or > 3500 kcal for women and < 800 kcal or > 4200 kcal for men),^[9]^ participants with missing data on KSD or adherence to the aMED score (n = 455), 28,059 participants were ultimately included in the analysis (Fig. 1).

### 2.2. Assessment of aMED and KSD

Initially, NHANES participants were interviewed face-to-face for 24 hours during the dietary recall. The second dietary recall was performed by telephone 3 to 10 days following the in-person assessment. Given the high response rate for the Day 1 dietary recall, we used the Day 1 dietary data for the primary analysis. Additionally, the average of the 2 days’ dietary data was used for the sensitivity analysis.^[8]^

The Mediterranean diet adherence was evaluated by converting 24-hour dietary recall data into equivalent units according to USDA food patterns.^[10]^ Then, following the method described by Gaskins et al.,^[11]^ We modified the aMED score for use with 24-hour dietary recall data.^[12]^ The aMED score comprises 9 elements: vegetables, whole grains, legumes, nuts, fruits, red and processed meats, fish, alcohol, and the monounsaturated to saturated fat ratio. The aMED score, ranging from 0 to 9, reflects the level of adherence to the MED, with higher scores indicating greater compliance (Table S1, Supplemental Digital Content, https://links.lww.com/MD/Q58). The primary independent variable was the quartiles of the aMED score.

The diagnosis of KSD was determined through the question, “Have you/the sample person ever had KSD?” Participants who responded “yes” were classified as having a history of KSD.

### 2.3. Covariates

We collected standardized questionnaires and measurements, adjusting for various covariates to strengthen our analysis. Demographic factors include age, gender, race, education level and poverty-to-income (PIR) ratio. Lifestyle factors include smoking, drinking, which categorized as: Never (fewer than 12 lifetime drinks), Ever (12 or more drinks in a single year or lifetime but not in the past year), and Now (currently consuming 12 or more drinks annually) and Physical activity levels were assessed based on their intensity, expressed as multiples of the task metabolic equivalent (MET), and classified into 2 groups: <500 MET-minutes per week and 500 or more MET-minutes per week. Health-related variables considered in the analysis included body mass index, hypertension, diabetes, cardiovascular disease (CVD), as well as self-reported histories of gout and cancer. For covariates with missing data exceeding 2%, corresponding dummy variables were created to account for these gaps.

### 2.4. Statistical analyses

We integrated 6 survey cycles utilizing complex sampling weights (MEC examination weight). Baseline characteristics were displayed in Table 1, We investigated the association between aMED score, analyzed as both a continuous and categorical variable (quartiles), and logistic regression was used to investigate the extent of the association between the aMED scores and KSD. additionally, trend tests were conducted accordingly. We utilized restricted cubic spline regression to analyze the dose-response relationship between aMED scores and KSD prevalence furthermore, we use the average of 2-day dietary recalls for sensitivity analysis. Finally, interaction tests were utilized to assess heterogeneity between subgroups. Statistical analysis was based on R 4.3.0 version, and *P* < .05 was considered statistically significant

**Table 1:** Characteristics of participants by aMED score quartiles: NHANES 2007 to 2018.

	All mean (95% Cl)	Q1 (0–2 pts)	Q2 (3 pts)	Q3 (4–5 pts)	Q4 (6–9 pts)
Age (year)	47.97 (47.49–48.45)	44.37 (43.82–44.92)	47.43 (46.76–48.11)	49.98 (49.34–50.62)	51.65 (50.76–52.54)
Age category (%)					
20–34	26.51 (25.15–27.88)	33.67 (32.19–35.15)	27.18 (25.37–28.99)	22.70 (21.01–24.40)	19.41 (17.41–21.42)
35–49	27.04 (25.45–28.63)	28.82 (27.60–30.05)	27.50 (25.90–29.11)	26.40 (24.85–27.95)	23.79 (21.60–25.99)
50–64	27.26 (25.64–28.88)	24.25 (23.09–25.41)	27.38 (25.81–28.95)	28.09 (26.48–29.69)	31.80 (29.34–34.26)
≥65	19.19 (17.89–20.48)	13.25 (12.20–14.31)	17.93 (16.67–19.20)	22.81 (21.59–24.03)	25.00 (22.85–27.15)
PIR	3.03 (2.96–3.10)	2.67 (2.59–2.76)	2.90 (2.83–2.98)	3.19 (3.10,3.27)	3.63 (3.53,3.73)
PIR category (%)					
≤1.3	19.49 (18.38–20.59)	25.55 (23.84–27.26)	21.29 (19.83–22.76)	16.46 (15.10–17.82)	10.55 (9.37–11.74)
1.3–3.5	33.13 (31.17–35.09)	35.78 (34.18–37.38)	33.74 (31.93–35.54)	32.61 (30.98–34.24)	27.22 (25.01–29.43)
>3.5	40.21 (37.27–43.15)	31.88 (29.81–33.95)	37.40 (35.40–39.41)	43.59 (41.22–45.95)	55.36 (52.55–58.18)
Missing	7.17 (6.51–7.83)	6.79 (5.97–7.61)	7.57 (6.62–8.51)	7.34 (6.39–8.30)	6.86 (5.72–8.00)
BMI (kg/m^2^)	29.10 (28.92–29.28)	30.05 (29.80–30.29)	29.47 (29.20–29.74)	28.77 (28.55–29.00)	27.13 (26.83–27.43)
BMI category (%)					
<25 kg/m^2^	29.35 (27.66–31.03)	25.32 (23.94–26.71)	27.15 (25.54–28.76)	29.86 (28.35–31.37)	41.54 (39.11–43.97)
25–30 kg/m^2^	32.92 (31.17–34.67)	31.31 (29.92–32.69)	32.34 (30.63–34.05)	34.08 (32.81–35.35)	34.40 (32.21–36.58)
≥30 kg/m^2^	37.73 (35.74–39.72)	43.37 (41.82–44.91)	40.51 (38.58–42.43)	36.06 (34.50–37.61)	24.06 (22.22–25.89)
Physical activity (MET-minutes/week)	4747.46 (4582.95–4911.97)	5745.95 (5435.96–6055.93)	5056 (4809.33–5302.66)	4248.38 (4069.75–4427.01)	3487.15 (3248.28–3726.01)
Physical activity (%)					
<500 MET-minutes/week	12.08 (11.35–12.81)	11.70 (10.81–12.59)	12.52 (11.60–13.43)	12.41 (11.50–13.33)	11.19 (9.76–12.63)
≥500 MET-minutes/week	65.74 (62.64–68.83)	63.33 (61.94–64.72)	64.04 (62.56–65.53)	65.89 (64.48–67.29)	74.20 (72.05–76.35)
Missing	22.18 (20.91–23.46)	24.97 (23.65–26.30)	23.44 (22.12–24.75)	21.70 (20.49–22.91)	14.60 (12.90–16.31)
Energy (Kcal)	2059.96 (2045.40–2074.53)	2039.76 (2015.05–2064.48)	2055.33 (2028.65–2082)	2063.76 (2043.34–2084.18)	2105.76 (2073.17–2138.34)
aMED score	3.50 (3.45–3.55)	1.55 (1.53–1.57)	3.00 (3.00–3.00)	4.41 (4.40–4.43)	6.39 (6.36–6.42)
Gender					
Female	52.49 (50.08–54.90)	48.18 (46.76–49.61)	50.71 (48.96–52.46)	54.55 (53.20–55.91)	59.99 (57.98–62.00)
Male	47.51 (45.32–49.70)	51.82 (50.39–53.24)	49.29 (47.54–51.04)	45.45 (44.09–46.80)	40.01 (38.00–42.02)
Race (%)					
Mexican American	8.27 (6.96–9.58)	7.97 (6.51–9.44)	8.78 (7.15–10.41)	8.76 (7.22–10.30)	6.60 (5.15–8.05)
Non-Hispanic Black	10.83 (9.65–12.00)	13.38 (11.46–15.30)	12.12 (10.33–13.91)	9.34 (8.17–10.50)	6.73 (5.53–7.93)
Non-Hispanic White	67.53 (62.19–72.88)	67.51 (64.34–70.68)	66.24 (63.02–69.46)	67.24 (64.38–70.10)	70.86 (68.02–73.69)
Other Hispanic	5.68 (4.84–6.52)	5.53 (4.40–6.66)	6.83 (5.61–8.06)	5.56 (4.65–6.48)	4.22 (3.44–5.01)
Other race	7.69 (6.91–8.47)	5.61 (4.91–6.31)	6.02 (5.09–6.96)	9.10 (7.93–10.27)	11.59 (9.91–13.26)
Marital (%)					
Divorced	10.22 (9.52–10.92)	11.62 (10.73–12.51)	10.78 (9.88–11.68)	9.46 (8.65–10.26)	8.12 (6.82–9.41)
Living with partner	7.92 (7.28–8.56)	10.21 (9.29–11.12)	7.97 (7.05–8.89)	6.75 (5.96, 7.55)	5.80 (4.50, 7.10)
Married	55.69 (52.38–59.01)	48.59 (46.78–50.41)	54.20 (52.15–56.26)	59.28 (57.45–61.11)	64.86 (62.06–67.67)
Never married	17.97 (16.85–19.09)	22.34 (20.64–24.04)	18.20 (16.49–19.90)	15.60 (14.26–16.94)	14.08 (12.25–15.91)
Separated	2.34 (2.11–2.57)	2.58 (2.17–2.99)	2.78 (2.24–3.32)	2.18 (1.84–2.52)	1.43 (0.93–1.93)
Widowed	5.86 (5.44–6.27)	4.66 (4.15–5.18)	6.08 (5.34–6.82)	6.73 (6.18–7.28)	5.71 (4.84–6.57)
Education (%)					
<9th grade	5.09 (4.58–5.59)	5.30 (4.64–5.95)	5.73 (5.02–6.43)	5.22 (4.59–5.85)	3.02 (2.43–3.62)
9–11th grade	10.06 (9.16–10.96)	14.11 (12.96–15.25)	10.64 (9.68–11.60)	8.16 (7.11–9.21)	4.92 (3.95–5.89)
High school graduate	22.99 (21.43–24.55)	28.49 (26.92–30.07)	25.93 (24.34–27.52)	20.25 (18.79–21.72)	12.45 (10.89–14.00)
Some college	31.36 (29.76–32.96)	33.30 (31.78–34.82)	32.27 (30.69–33.85)	30.77 (29.07–32.47)	26.75 (24.73–28.77)
College or above	30.50 (27.99–33.02)	18.80 (17.17–20.44)	25.44 (23.20–27.68)	35.60 (33.26–37.94)	52.86 (49.98–55.73)
Smoke (%)					
Former	24.91 (23.18–26.64)	21.91 (20.71–23.10)	24.42 (22.64–26.20)	26.44 (24.91–27.97)	28.51 (26.40–30.61)
Never	56.18 (53.74–58.62)	48.70 (47.01–50.39)	55.77 (54.06–57.47)	59.97 (58.33–61.61)	63.64 (61.36–65.92)
Now	18.91 (17.73–20.08)	29.40 (27.79–31.00)	19.81 (18.43–21.19)	13.59 (12.60–14.58)	7.86 (6.55–9.16)
Alcohol (%)					
Former	11.79 (10.84–12.75)	13.36 (12.18–14.55)	11.94 (10.83–13.04)	11.62 (10.78–12.46)	8.30 (7.05–9.54)
Never	10.07 (9.15–10.99)	8.90 (8.12–9.68)	10.91 (9.60–12.22)	10.95 (9.91–11.99)	8.71 (7.32–10.11)
Now	70.48 (66.95–74.01)	70.08 (68.40–71.76)	70.28 (68.49–72.08)	69.03 (67.39–70.68)	76.07 (73.84–78.31)
Miss	7.66 (7.05–8.27)	7.66 (6.87–8.44)	6.88 (6.08–7.67)	8.39 (7.43–9.35)	6.92 (5.73–8.11)
Diabetes (%)					
No	77.22 (73.61–80.82)	78.07 (76.68–79.45)	75.77 (74.40–77.15)	76.12 (74.89–77.34)	81.12 (79.49–82.75)
Yes	22.78 (21.53–24.03)	21.93 (20.55–23.32)	24.23 (22.85–25.60)	23.88 (22.66–25.11)	18.88 (17.25–20.51)
Hypertension (%)					
No	61.78 (58.81–64.74)	62.74 (61.41–64.07)	60.42 (58.82–62.03)	60.42 (58.91–61.93)	66.00 (63.61–68.40)
Yes	38.22 (36.26–40.19)	37.26 (35.93–38.59)	39.58 (37.97–41.18)	39.58 (38.07–41.09)	34.00 (31.60–36.39)
CVD (%)					
No	91.14 (87.01–95.27)	91.51 (90.80–92.22)	90.75 (89.86–91.63)	90.82 (90.07–91.57)	91.94 (90.83–93.04)
Yes	8.86 (8.24–9.48)	8.49 (7.78–9.20)	9.25 (8.37–10.14)	9.18 (8.43–9.93)	8.06 (6.96–9.17)
Gout (%)					
No	95.87 (91.61–100.14)	95.63 (94.92–96.33)	96.01 (95.33–96.68)	95.80 (95.29–96.31)	96.44 (95.67–97.20)
Yes	4.13 (3.74–4.51)	4.37 (3.67–5.08)	3.99 (3.32–4.67)	4.20 (3.69–4.71)	3.56 (2.80–4.33)
Cancer (%)					
No	89.31 (85.40–93.21)	91.19 (90.43–91.94)	89.27 (88.13–90.42)	88.84 (87.95–89.73)	86.24 (84.68–87.80)
Yes	10.69 (9.95–11.44)	8.81 (8.06–9.57)	10.73 (9.58–11.87)	11.16 (10.27–12.05)	13.76 (12.20–15.32)
KSD (%)					
No	89.92 (85.94–93.89)	89.48 (88.49–90.48)	89.27 (88.10–90.43)	90.00 (89.12–90.87)	91.92 (90.55–93.28)
Yes	10.08 (9.39–10.77)	10.52 (9.52–11.51)	10.73 (9.57–11.90)	10.00 (9.13–10.88)	8.08 (6.72–9.45)

BMI = body mass index, CVD = cardiovascular disease, NHANES = National Health and Nutrition Examination Survey, PIR = poverty income ratio.

For continuous variables: survey-weighted mean (95% CI). For categorical variables: survey-weighted percentage (95% CI).

## 3. Results

### 3.1. Participants’ baseline characteristics

Table 1 classifies participants according to quartiles of the aMED score, encompassing a total of 28,059 adults, with a weighted average age (95% CI) of 47.97 (47.49, 48.45) years. Among them, 52.49% (50.08%, 54.90%) were male and 47.51% (45.32%, 49.70%) were female, with an overall KSD prevalence of 10.08% (95% CI: 9.39%, 10.77%). Higher aMED scores were associated with a decreased prevalence of KSD.

### 3.2. Multivariate regression analysis

**Figure 2.**
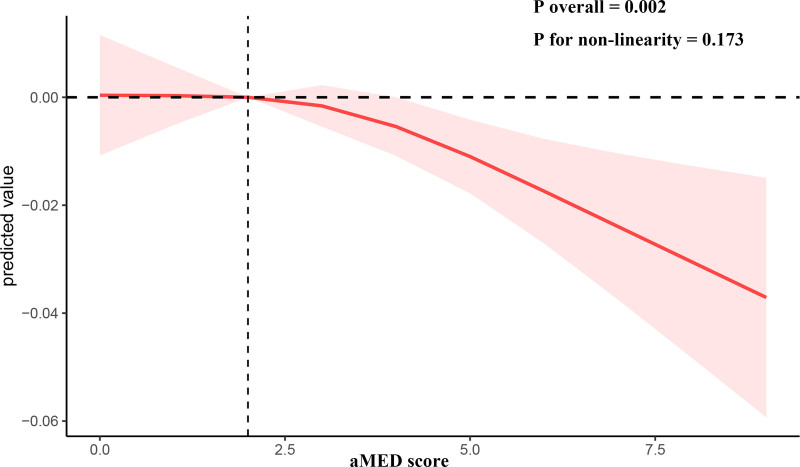
The RCS of the association between aMED score and KSD. aMED = alternate Mediterranean diet score, KSD = kidney stone disease.

Multivariate regression analysis indicated a negative correlation between aMED scores and KSD prevalence across all models: Model 1 (OR = 0.996, 95% CI 0.993–0.999, *P* = .005), Model 2 (OR = 0.993, 95% CI 0.99–0.996, *P* < .0001), and Model 3 (OR = 0.996, 95% CI 0.993–0.999, *P* = .013). Q4 showed a negative association with KSD incidence across all 3 models. In the fully adjusted model, the probability of developing KSD in Q4 was 22.9% lower than that in Q1 (OR = 0.771, 95% CI: 0.616 to 0.966, *P* = .024).The *P*-value for trend was < 0.001 (Table 2).Multivariate regression analysis indicated that total fruit products (OR 0.986, 95% CI 0.975–0.996, *P* = .007) and whole grain products (OR 0.986, 95% CI 0.997–0.997, *P* = .013) were linked to KSD risk in the fully adjusted model (Table 3). The restricted cubic spline model, adjusted for covariates, demonstrated a linear inverse relationship between aMED scores and the risk of KSD (Fig. 2).

**Table 2:** Association of aMED score with KSD.

Exposure	OR (95% CI), *P*-value		
	Model 1*	Model 2†	Model 3‡
aMED (continuous)	0.996 (0.993–0.999) **.005**	0.993 (0.99–0.996) < **.0001**	0.996 (0.993–0.999) **.013**
aMED (in quartiles)			
Quartile 1	Ref	Ref	Ref
Quartile 2	1.023 (0.848–1.235) .810	0.959 (0.794–1.160) .666	0.999 (0.825–1.210) .992
Quartile 3	0.946 (0.819–1.093) .446	0.837 (0.724–0.967) **.017**	0.910 (0.785–1.054) .204
Quartile 4	0.748 (0.610–0.919) **.006**	0.636 (0.515–0.785) < **.0001**	0.771 (0.616–0.966) **.024**
*P* for trend	**.005**	**<.0001**	**.014**

Statistically significant values were reported in bold.

aMED = alternate Mediterranean diet score, BMI = body mass index, CI = confidence interval, CVD = cardiovascular disease, OR = odds ratio, PIR = poverty income ratio.

* Non-adjusted model: adjusted for none.

† Minimally adjusted model: adjusted for gender, age, race.

‡ Fully adjusted model: adjusted for gender, age, race, PIR, BMI, education, marital status, smoking, alcohol, energy, physical activity, gout, diabetes, hypertension, stroke, CVD and cancer.

**Table 3:** Relationship between aMED score components and KSD.

aMED component	OR (95% CI), *P*-value		
	Model 1*	Model 2†	Model 3‡
Vegetables	0.998 (0.986–1.009) .673	0.991 (0.979–1.003) .157	0.996 (0.984–1.008) .539
Legumes	0.993 (0.981–1.006) .306	0.996 (0.984–1.009) .538	1.001 (0.988–1.015) .824
Fruits	0.986 (0.977–0.995) **.004**	0.978 (0.968–0.987) < **.0001**	0.986 (0.975–0.996) **.007**
Nuts	0.997 (0.986–1.007) .538	0.988 (0.977–0.999) **.029**	0.994 (0.982–1.006) .295
Whole grains	0.994 (0.984–1.003) .210	0.982 (0.972–0.992) < **.001**	0.986 (0.975–0.997) **.013**
Red and processed meats	0.999 (0.989–1.009) .862	1.001 (0.991–1.010) .910	1.001 (0.991–1.011) .838
Fish	0.992 (0.980–1.004) .194	0.992 (0.980–1.004) .183	0.995 (0.982–1.008) .420
Alcohol	1.005 (0.982–1.027) .679	0.999 (0.978–1.021) .950	1.012 (0.990–1.034) .288
Ratio of monosat lipid to sat lipid	0.992 (0.983–1.001) .072	0.993 (0.984–1.002) .109	0.993 (0.984–1.003) .157

Statistically significant values were reported in bold.

aMED = alternate Mediterranean diet score, BMI = body mass index, CI = confidence interval, CVD = cardiovascular disease, OR = odds ratio, PIR = poverty income ratio.

* Non-adjusted model: adjusted for none.

† Minimally adjusted model: adjusted for gender, age, race.

‡ Fully adjusted model: adjusted for gender, age, race, PIR, BMI, education, marital status, smoking, alcohol, energy, physical activity, gout, diabetes, hypertension, stroke, CVD and cancer.

### 3.3. Stratified and sensitivity analysis

**Figure 3.**
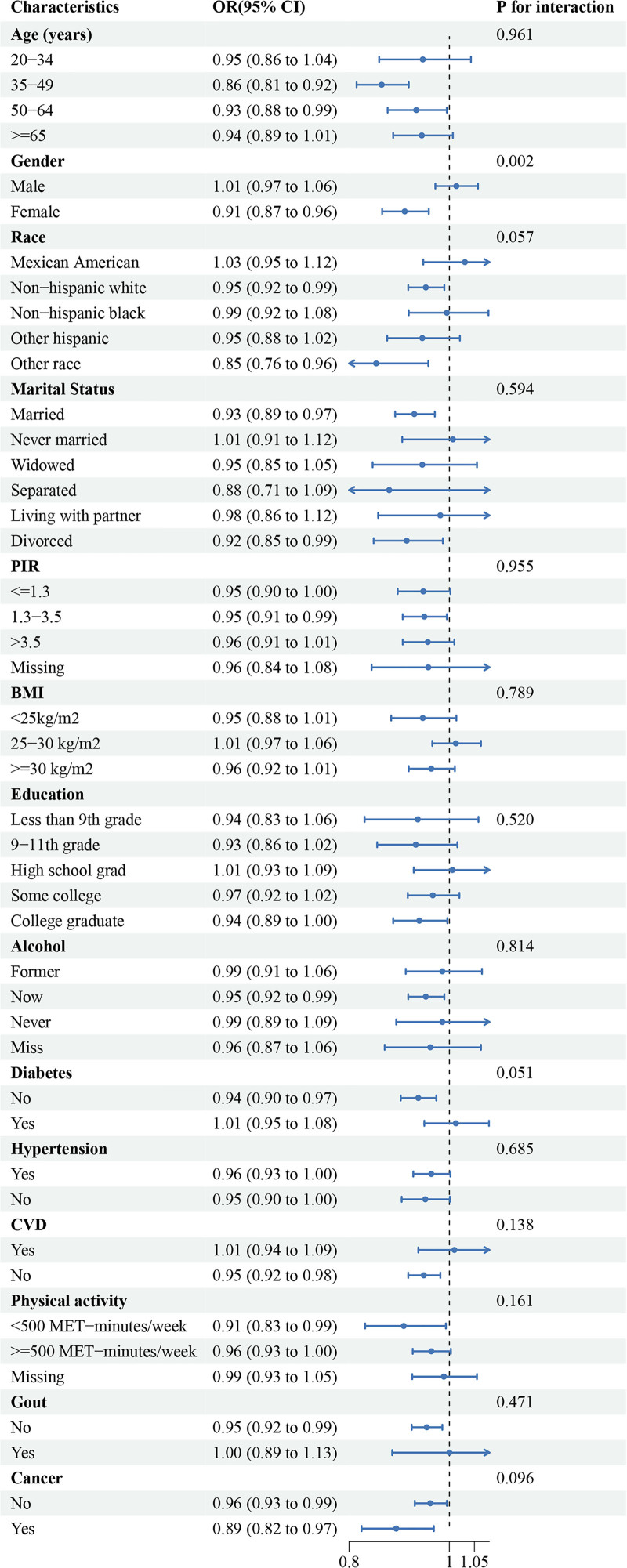
The stratified logistic regression analysis.

Subgroup analysis indicated that gender modified the effect of aMED scores on KSDs, with an interaction *P*-value of 0.02, after adjusting for other covariates. Detailed information about stratified analysis (Fig. 3). Sensitivity analysis using the average dietary intake over 2 days in multinomial logistic regression similarly indicated a negative correlation between aMED scores and the incidence of KSD, with statistically significant differences observed in the highest aMED quartile (Q4) (*P* < .05) (Table S2, Supplemental Digital Content, https://links.lww.com/MD/Q58).

## 4. Discussion

We found that individuals who strictly followed the MED had a 22.8% to 32.3% lower risk of developing KSD compared to the lowest quantile. This finding provides strong support for the role of dietary interventions in preventing the occurrence of KSD.^[13]^

Prior research indicates that following the MED significantly lowers the risk of metabolic diseases.^[14,15]^ Metabolic abnormalities are a known risk factor for KSD, and dietary modifications to enhance metabolic health are deemed an effective strategy for their prevention and treatment.^[16]^ A previous cross-sectional study found that an elevated healthy eating index significantly lowered the risk of KSD, especially with increased fruit and vegetable intake.^[17]^ The DASH diet has been shown to reduce KSD incidence, likely due to its focus on limiting sodium intake.^[18]^

Our research indicates that a higher aMED score is significantly associated with a lower prevalence of KSD. The MED, rich in fiber from fruits, vegetables, whole grains, and healthy fats, with moderate fish intake, limits red meat and processed foods. This dietary pattern may lower KSD risk through several physiological mechanisms. Red meats, like beef and lamb, are high in purines, which break down into uric acid, increasing the likelihood of uric acid stones. Processed foods, often high in refined sugars and high-fructose corn syrup, can raise urinary levels of calcium, oxalate, and uric acid, contributing to stone formation.^[19]^ In contrast, the healthy fats in the MED, primarily from olive oil and nuts, are rich in monounsaturated (MUFA) and polyunsaturated fatty acids (PUFA), particularly omega-3s. These fats have anti-inflammatory effects and help improve lipid metabolism. Additionally, fish high in omega-3s and vitamin D support calcium metabolism and reduce inflammation, further lowering the risk of KSD.^[20]^

Component analysis indicated a significant protective link with high consumption of fruits and whole grains. Dietary fiber from fruits and whole grains can bind dietary oxalate in the intestinal lumen and increase fecal oxalate excretion, thereby reducing intestinal oxalate absorption and subsequent urinary oxalate excretion; high-fiber diets also accelerate intestinal transit and promote a gut microbiome favorable to oxalate-degrading bacteria (e.g., Oxalobacter formigenes), together lowering systemic oxalate load and the risk of oxalate stones.^[21]^ In addition, greater fiber intake is associated with improved metabolic profiles (better insulin sensitivity and weight control), which may reduce endogenous uric acid production and improve renal uric acid handling, plausibly lowering the likelihood of uric acid stones. Fruits are also rich in potassium, citrate precursors and antioxidants (such as vitamin C and polyphenols), which can increase urinary citrate, mitigate oxidative stress and inflammation, and thereby create a urinary environment less conducive to crystal formation.^[22]^ Additionally, whole grains are high in potassium, which can raise urinary pH and reduce calcium excretion, playing a crucial role in preventing calcium stone formation.^[23]^ The aMED score also takes moderate alcohol consumption into account. Moderate alcohol intake may offer some protection against certain types of stones, such as calcium stones, as alcohol acts as a diuretic, increasing urine production and diluting the concentration of minerals in the urine. Furthermore, moderate red wine consumption, which is rich in polyphenolic antioxidants, may help reduce oxidative stress.^[24,25]^

Stratified analysis revealed an interaction between gender differences and aMED scores in relation to KSD risk. This may be attributed to significant differences between men and women in hormone levels, metabolism, and lifestyle factors, all of which directly influence the incidence of KSD. Estrogen is thought to protect women from calcium stone formation by enhancing bone calcium deposition and decreasing urinary calcium excretion. Estrogen may decrease urinary calcium excretion by enhancing the activity of renal sodium-hydrogen exchangers.^[26]^ Furthermore, men tend to have higher rates of alcohol consumption and smoking, behaviors that are less prevalent among women. Women’s naturally higher urinary pH also helps prevent the formation of uric acid stones.^[27]^

Our study possesses multiple strengths. First, the use of nationally representative data enhances the credibility and generalizability of our findings. Second, the use of the aMED score, an internationally recognized dietary assessment tool, is more reliable compared to single dietary indicators. Finally, we performed sensitivity analysis and stratified analysis to verify the reliability of the results in the correction of confounding conditions.

Importantly, these findings have significant public health implications. Promoting adherence to Mediterranean dietary patterns through dietary counseling in primary care settings and broader public health campaigns may be a feasible, cost-effective strategy to reduce the burden of KSD. By integrating dietary guidance emphasizing fruits, whole grains, and healthy fats, healthcare providers can support patients in adopting lifestyle changes that potentially lower KSD prevalence and improve overall metabolic health.

Nonetheless, our research presents certain limitations. As this is a cross-sectional study, a direct causal link between the aMED score and KSD formation cannot be established. The dietary and KSD data relied on participants’ recall and questionnaire responses, potentially introducing recall and reporting biases. Ultimately, we could not differentiate between various KSD types, including uric acid and calcium oxalate stones.

## 5. Limitations

This study has several limitations. First, dietary intake was primarily assessed using a single 24-hour recall, which may not accurately reflect usual or long-term dietary habits. Although sensitivity analyses using the average of 2 recall days were conducted, the limited number of dietary assessment days could lead to measurement error and attenuation of observed associations. Second, KSD status was self-reported via a binary question about “ever having” kidney stones, without clinical verification such as imaging or medical records. This self-report method may be subject to recall bias and misclassification, potentially impacting the accuracy of KSD prevalence estimates. Third, this is a cross-sectional study, and therefore no causal or temporal relationship between aMED adherence and KSD risk can be inferred. Future prospective studies with repeated dietary assessments and clinically confirmed KSD diagnoses are warranted to further validate and expand upon these findings.

## 6. Conclusion

In U.S. adults, an increased aMED score correlates with a reduced prevalence of KSD, demonstrating a negative linear relationship. However, further large-scale, multicenter studies are needed to establish a causal relationship.

## Author contributions

**Conceptualization:** Jian-wei Yang, Xiao-ran Li, Li Yang.

**Data curation:** Jian-wei Yang, Li Yang.

**Formal analysis:** Si-yu Chen, Li Wang, Jian-wei Yang, Kang-yu Wang, Li Yang.

**Investigation:** Si-yu Chen, Li Wang.

**Methodology:** Si-yu Chen, Li Wang, Kang-yu Wang.

**Project administration:** Li Wang, Kang-yu Wang, Xiao-ran Li, Li Yang.

**Resources:** Li Wang.

**Supervision:** Xiao-ran Li.

**Visualization:** Li Yang.

**Writing – original draft:** Xiao-ran Li, Li Yang.

**Writing – review & editing:** Xiao-ran Li, Li Yang.

## Supplementary Material


