# Supplementary material for: Mediterranean diet adherence and risk of kidney stones: Insights from a population-based study
Source: Medicine (Baltimore). 2025 Sep 19;104(38):e44653. doi: 10.1097/MD.0000000000044653 (PMC12459513; doi:10.1097/MD.0000000000044653)
Supplement: Supplementary file 1 [file medi-104-e44653-s001.docx]

**Table S1.** The detail of aMED score

| **Food Groups** | **Description** | **Units** | **Minimum score: 0** | **Maximum score: 1** |
| --- | --- | --- | --- | --- |
| Whole Fruit + Fruit juice | All fruits and 100% fruit juices | servings/day (0.5 c of berries; 1 cup=236.59 g; 1 med fruit (1 cup = 236.59 g); 1 cup fruit juice | < Median | ≥ Median |
| Vegetable | All vegetables except potatoes and legumes | 0.5 c of vege; 1 cup of green leafy | < Median | ≥ Median |
| Whole grain product | Whole-grain ready-to-eat cereals, cooked cereals, crackers, dark breads, brown rice, other grains, wheat germ, bran, popcorn | 1oz | < Median | ≥ Median |
| Legume | Tofu, string beans, peas, beans | *oz, 1 cup legume = 4 oz | < Median | ≥ Median |
| Nut | Nuts, peanut butter | *Oz equiv | < Median | ≥ Median |
| Fish | Fish and shrimp, breaded fish | serving (4 oz) | < Median | ≥ Median |
| Red and processed meat | Beef, pork, lamb, goat, veal, sausages, bacon, salami, ham, hot dog, deli meat, organ meat | serving (4 oz. unprocessed meat; 1.5 oz. processed meat) | ≥ Median | < Median |
| Ratio of monosat lipid to sat lipid | N/A | Ratio | < Median | ≥ Median |
| Alcohol | Wine, beer, "light" beer, liquor | g | < 10 or >25 g/d | 10-25 g/day |
|  |  | **Total possible score** | **0** | **9** |

Reference(s):

1. Fung T T, Rexrode K M, Mantzoros C S, et al. Mediterranean diet and incidence of and mortality from coronary heart disease and stroke in women[J]. Circulation, 2009, 119(8): 1093-1100.

2. Chiuve SE, Fung TT, Rimm EB, Hu FB, McCullough ML, Wang M, Stampfer MJ, Willett WC. Alternative dietary indices both strongly predict risk of chronic disease. J Nutr. 2012 Jun;142(6):1009-18. doi: 10.3945/jn.111.157222. Epub 2012 Apr 18. PMID: 22513989; PMCID: PMC3738221.

**Table S2** Association of Alternate Mediterranean Diet (aMED) score (mean of two days) with kidney stones (day 2 dietary recall)

| Exposure | OR (95%CI), *P* value | | |
| --- | --- | --- | --- |
|  | Model 1^a^ | Model 2^b^ | Model 3^c^ |
| aMED (continuous) | 0.994 (0.991, 0.998) **0.002** | 0.99 (0.986,0.994) **<0.0001** | 0.993 (0.988, 0.998) **0.006** |
| aMED (in quartiles) |  |  |  |
| Quartile 1 | Ref | Ref | Ref |
| Quartile 2 | 0.991(0.851,1.153) 0.905 | 0.912(0.778,1.070) 0.254 | 0.926(0.775,1.106) 0.391 |
| Quartile 3 | 0.974(0.826,1.147) 0.747 | 0.850(0.714,1.012) 0.068 | 0.891(0.727,1.092) 0.26 |
| Quartile 4 | 0.748(0.628,0.892) **0.002** | 0.627(0.522,0.753) **<0.0001** | 0.721(0.577,0.901) **0.005** |
| *P* for trend | **0.003** | **<0.0001** | **0.007** |

OR, odds ratio; CI: confidence interval; aMED, Alternate Mediterranean Diet score; PIR, poverty income ratio; BMI, body mass index; CVD, cardiovascular disease;

^a^Non-adjusted model: adjusted for none.

^b^Minimally adjusted model: adjusted for gender, age, race.

^c^Fully adjusted model: adjusted for gender, age, race, PIR, BMI, education, marital status, smoking, alcohol, energy, physical activity, gout, diabetes, hypertension, stroke, CVD and cancer

Statistically significant values were reported in bold
